# Modelling Friction Reduction Based on Molybdenum Disulphide Tribofilm Formation and Removal in Boundary Lubrication

**DOI:** 10.1007/s11249-025-01981-6

**Published:** 2025-03-17

**Authors:** Dichu Xu, Cayetano Espejo, Chun Wang, Ardian Morina

**Affiliations:** 1 Ningbo Institute of Technology, Beihang University, Ningbo, 315800 China; 2 Institute of Functional Surfaces, School of Mechanical Engineering, University of Leeds, Leeds, LS29JT UK

**Keywords:** Boundary lubrication, MoS_2_ tribofilm, Friction, Tribochemical reaction

## Abstract

Molybdenum dialkyldithiocarbamate is a highly effective friction modifier lubricant additive in boundary lubrication, owing to the formation of a MoS_2_ nanosheet lattice structure that significantly reduces friction. The friction reduction behaviour is linked to the MoS_2_ amount and coverage buildup at the contacting interface, however, accurately predicting friction reduction based on a semi-deterministic model incorporating MoS_2_ formation and removal remains challenging. In this study, a Raman map collection methodology was developed for accurate quantitative analysis of MoS_2_ tribofilms. The growth rate of MoS_2_ tribofilms was determined by coupling tribochemical experimental data with sophisticated numerical models. A full numerical procedure was implemented under rubbing of two rough surfaces at different temperatures. The results demonstrated localised MoS_2_ tribofilms buildup. The friction coefficients show a close agreement with the measurements. The developed model can be adapted to diverse experimental setups and surface geometries.

**Supplementary Information:**

The online version contains supplementary material available at 10.1007/s11249-025-01981-6.

## Introduction

Automotive engine manufacturers are facing significant challenges in increasing vehicle fuel economy and reducing carbon emissions. It is well-established that the use of lubricant additives can significantly reduce friction losses, lower fuel consumption, and maintain durability [1–3]. In this regard, predicting the performance of lubricant additives in engine tribology systems has become one of the most important research areas for the development of optimized energy-efficient systems [4–7].

In the boundary lubrication regime, interactions between the surface and lubricant lead to the formation of an ultra-thin, reactive layer known as a “tribofilm”. It modifies the metal–metal interface and plays a crucial role in reducing wear and friction at the interface. Although the tribofilm build-up is experimentally well researched, leading to a good understanding of tribofilm chemical structure and compounds [4, 8, 9], progress in predicting the complex processes of tribofilm formation through numerical models remains limited.

Compared to conventional thermochemical reactions, the rubbing between contacting surfaces can significantly enhance the activation of chemical reactions to form tribochemical layers at the interface. Hence, the simulation of the activation of chemical reactions through tribocontacts has garnered increased attention, particularly for its significance in the boundary lubrication regime [10–12]. Gosvami et al. constructed an equation to express the stress-dependent growth rate based on molecular-level experiments [13]. Just as the growth rate depends on temperature, they found that variations in growth rate with contact pressure follow an exponential pattern as well. Zhang and Spikes developed a stress-promoted thermal activation model and showed that ZDDP film formation could be driven by shear stress in the elastohydrodynamic lubrication (EHL) fluid films [14, 15]. Bulgarevich et al. modified the Arrhenius equation by introducing a correction factor representing the mechanical rubbing on the tribochemical reactions [16, 17]. Based on that, Ghanbarzadeh et al. proposed a wear model with consideration of the effect of ZDDP tribofilm on the reduction in wear, and good agreements have been shown between wear measurements and simulation results [18]. Akchurin and Bosman used a stress-activation Arrhenius model combined with a mild wear tribofilm model to simulate evolution of ZDDP tribofilm thickness [19]. Azam et al. employed experimental data fitting techniques to simulate the growth and removal of local tribofilm thickness [20]. Gong et al. simulated wear evolution by modelling the tribochemical wear coefficient which was assumed to be related to the thickness and composition of tribofilms [7].

The key variable captured by the previous experiments and numerically studied by the above-mentioned models is the physical thickness of the integrated antiwear tribofilms (e.g. antiwear ZDDP tribofilm). In these models, the thickness of the local tribofilm is critical for determining whether solid–solid contact between asperities occurs, which in turn influences the loss of substrate material and allows for the prediction of wear rates. Among friction modifier lubricant additives, molybdenum dialkyldithiocarbamate (MoDTC) provides excellent friction behavior in boundary lubricated tribocontacts. It is well-established that the low friction obtained with MoDTC is due to the formation of lattice structure MoS_2_ nanosheets. However, the overall thickness of the integrated tribofilm cannot serve as a predictive measure for friction reduction when the modification is attributed to MoS_2_. Instead, friction reduction is linked to the development of the MoS_2_ tribofilm's crystalline structure [21, 22], with MoS_2_ dispersing within the tribofilm matrix—a phenomenon that can be observed in transmission electron microscopy (TEM) images [23, 24]. Thus, the extent of friction reduction is more closely related to the presence and distribution of MoS_2_ within the tribofilm rather than its thickness [25].

Molecular dynamics (MD) simulations, particularly those utilizing reactive force fields such as ReaxFF, have recently been developed to investigate the dynamics of tribochemical reactions leading to MoS_2_ formation [26–28]. These reactive MD simulations provide valuable insights into tribochemical reactions and mechanisms of friction reduction at an atomistic level by allowing for the dynamic formation and breaking of chemical bonds during tribological interactions. While MD simulations effectively model individual asperity contacts at the atomistic level, they are constrained in addressing collective asperity interactions and micron-scale friction phenomena. Consequently, there remains a need for mesoscopic friction models that incorporate tribochemistry. Additionally, the MoS_2_ tribofilm is prone to easy shearing, undergoing continuous formation and removal through contact cycles. Therefore, it is crucial to account for the dynamic processes of MoS_2_ tribofilm formation and removal, as the tribochemical equilibrium achieved promotes low friction and mild wear in a steady-state.

This study aims to develop a semi-deterministic friction model capable of capturing the dynamic growth of the MoS_2_ tribofilm on the MoDTC/ZDDP tribofilm surface, building upon the authors' prior experimental investigations into the characterization of MoS_2_ tribofilms [25]. To accurately quantify the growth rates of MoS₂ tribofilms, Raman mapping data from prior experiments were re-examined, and a refined Raman mapping methodology was developed to ensure reliable, quantitative analyses across the wear scar. This work led to the creation of a calibration procedure that integrates these experimental measurements with advanced numerical modelling tools, allowing for more precise parameter refinement. The resulting model was rigorously validated against experimental data, demonstrating its robust capability to predict friction performance across a range of temperature conditions. By incorporating tribochemical dynamics, the numerical model provides deeper insights into the mechanisms driving MoS₂ tribofilm formation and evolution, offering a clearer understanding of their influence on friction behaviour from the microscale to the macroscale.

## Raman Map Calibration

To determine the growth rate of MoS_2_ tribofilm, as a first step, the quantity of the MoS_2_ tribofilm concentration within the wear scar needs to be estimated. Raman mapping is demonstrated to be a powerful technique to detect the quantity and spatial distribution of MoS_2_ tribofilms across the wear scar [25, 29]. The methods for obtaining the Raman maps significantly influence the quantitative analysis of MoS_2_ tribofilms. Thus, in the first part of this paper, the results of previous Raman mapping analyses will be re-examined to address the following questions: (1) how is the scale of the Raman map chosen to be a good representative of MoS_2_ tribofilm distribution? (2) how many Raman maps should be collected along the wear scar to achieve an acceptable low sampling error? (3) how many spectra need to be collected in one Raman map to obtain a reliable quantitative analysis? The discussions on these questions will ensure the accuracy and precision of quantitative analysis of Raman maps on the MoS_2_ tribofilm concentration and provide a strong premise for the following numerical modelling.

### Effect of Directionality

As in previous experimental investigation, the Raman measurements were performed using a Renishaw InVia spectrometer equipped with a 488 nm laser excitation source [25](Page 13,524). The laser power was carefully controlled at 1 mW to minimize sample heating and prevent potential damage to the tribofilm. Measurements were conducted with a 50 × objective lens, achieving a spatial resolution of 800 nm. Each Raman spectrum was acquired with an exposure time of 20 s to ensure an adequate signal-to-noise ratio. Baseline correction was applied to each spectrum to remove background signals using a third-order polynomial fitting method. MoS₂ tribofilms were characterized through normalized Raman intensity mapping of the A₁_g_ symmetry mode at 412 cm^−1^, where normalization was performed relative to the maximum peak intensity. Any amorphous MoSₓ compounds would be expected to appear as a Raman peak at a different mode to the peak at 412 cm^−1^ used in this study. Based on the Raman results, their influence was found to be negligible. Under the specified experimental conditions, the Raman spectra predominantly reflect the crystalline MoS₂ phase, ensuring that the observed A₁_g_ peak intensity is primarily associated with the desired tribofilm formation.

**Fig. 1:**
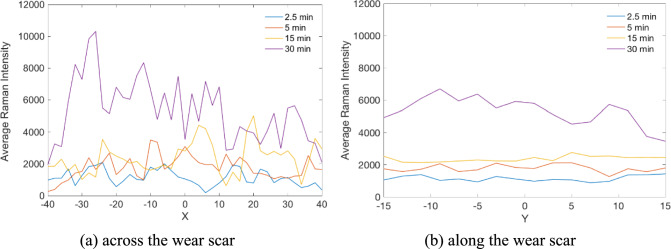
Average Raman intensities from original Raman maps of 80 × 30 μm^2^ at various rubbing time. The data are averaged from a set of spectra, a single row taken from the Raman map in the Y (or X) direction **a** across the wear scar **b** along the wear scar

According to the representative Raman maps, the MoS_2_ tribofilms were heterogeneously distributed across the wear scar [25](Page 13,527). To quantitatively investigate the spatial distribution features of the MoS₂ tribofilm, the Raman maps were subdivided into a single row of data. For each spectrum, the intensity of the A₁_g_ peak, which is characteristic of MoS₂, was extracted as a representative parameter of the tribofilm. These intensities were then averaged across the wear scar (see Fig. 1a) and along the wear scar (see Fig. 1b, where the Y-axis represents the direction of sliding), respectively. The “average Raman intensity” thus reflects the spatial distribution of the A₁_g_ peak height, providing an overview of the tribofilm’s heterogeneity and offering a representative parameter to assess the quality and uniformity of the Raman maps. Figure 1a shows more randomly dispersed and fluctuating data, while the sequence shown in Fig. 1b tends to be more uniform. This strongly suggests that the distribution of the MoS_2_ tribofilms across the wear scar is more heterogeneous than along the wear scar. Thus, how the length scale would be selected across the wear scar makes a great difference to the calibration of MoS_2_ concentration. In contrast, the variation in the MoS_2_ concentration along the wear scar is less critical. It can also be concluded that appropriately reducing the Y dimension of the Raman map can optimize instrument usage time and potentially protect samples from laser-induced damage.

### Effect of Raman Map Dimension

**Fig. 2:**
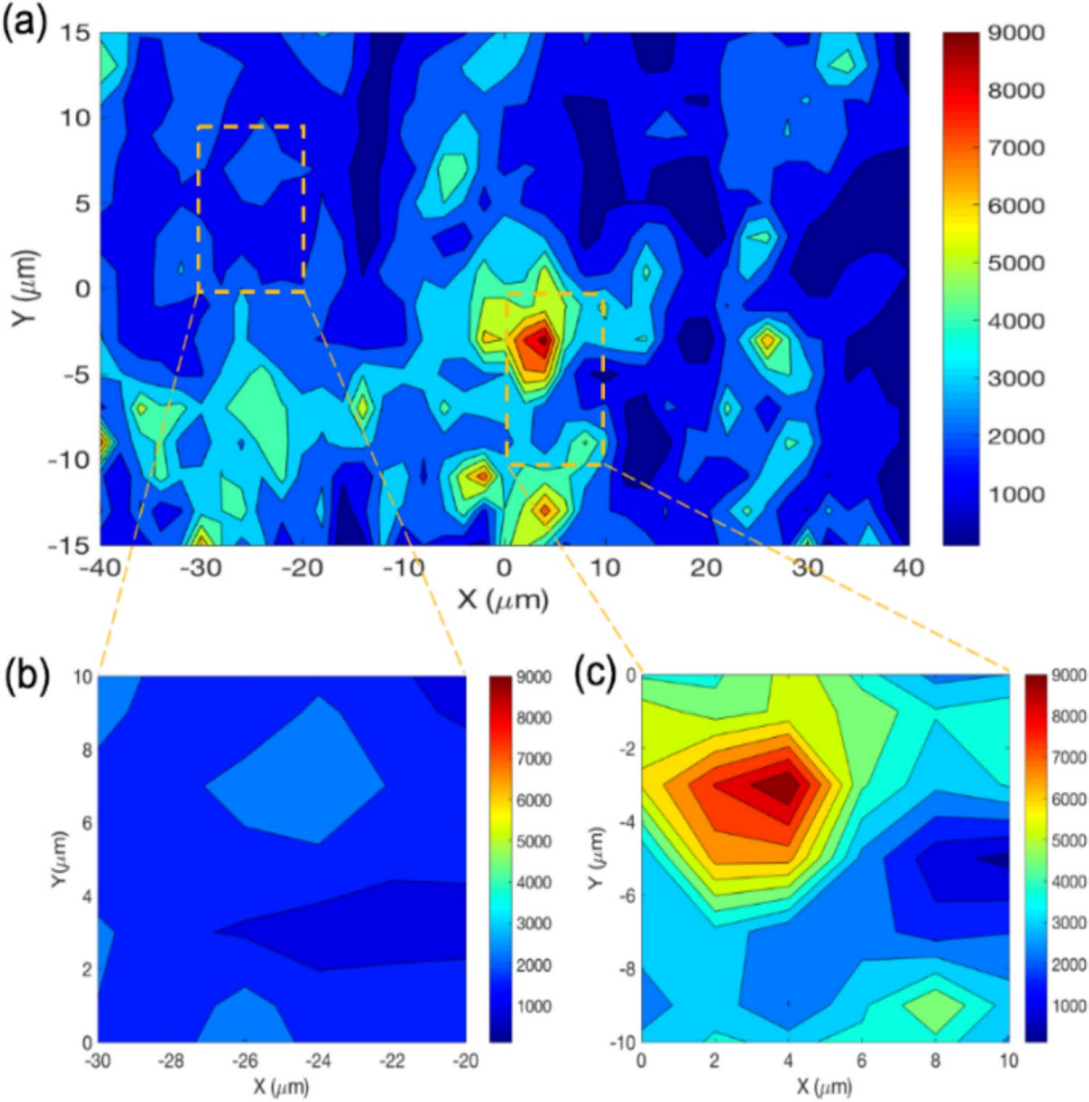
Raman mapping characteristics after 15 min of rubbing at 80 °C: **a** Raman map as a typical example of what was presented in publication [25] **b** sub-level Raman map from **c** with low concentration **d** sub-level Raman map from **e** with high concentration

Figures 2b and c show two micro-scale Raman maps (10 × 10 μm^2^) were taken at different locations in the larger Raman map (Fig. 2a) of 80 × 30 μm^2^. Figures 2b and c exhibit very different MoS_2_ concentrations, which indicates the heterogeneity of MoS_2_ tribofilm distribution across the wear scar. In this work, the use of Raman maps is considered for quantitative calibration of the tribochemistry model, and to assess the scale of the map that should be used for the most accurate overall prediction of friction.

In general, it would be expected that the largest Raman mapping area should give the more realistic integrated MoS_2_ concentration in the wear scar, and more Raman maps measured along the wear scar will reduce the sampling errors. However, full mapping of the whole wear scar is impractical since there is a risk of burning the sample surface with long-time laser exposure. The alternative way is to sample the overall wear scar by combining Raman intensity maps taken at different locations across the overall wear scar. The questions here are: how to determine the appropriate dimension of the Raman map and how many Raman maps should be collected to be a good representative of MoS_2_ concentration and achieve low sampling errors?

**Fig. 3:**
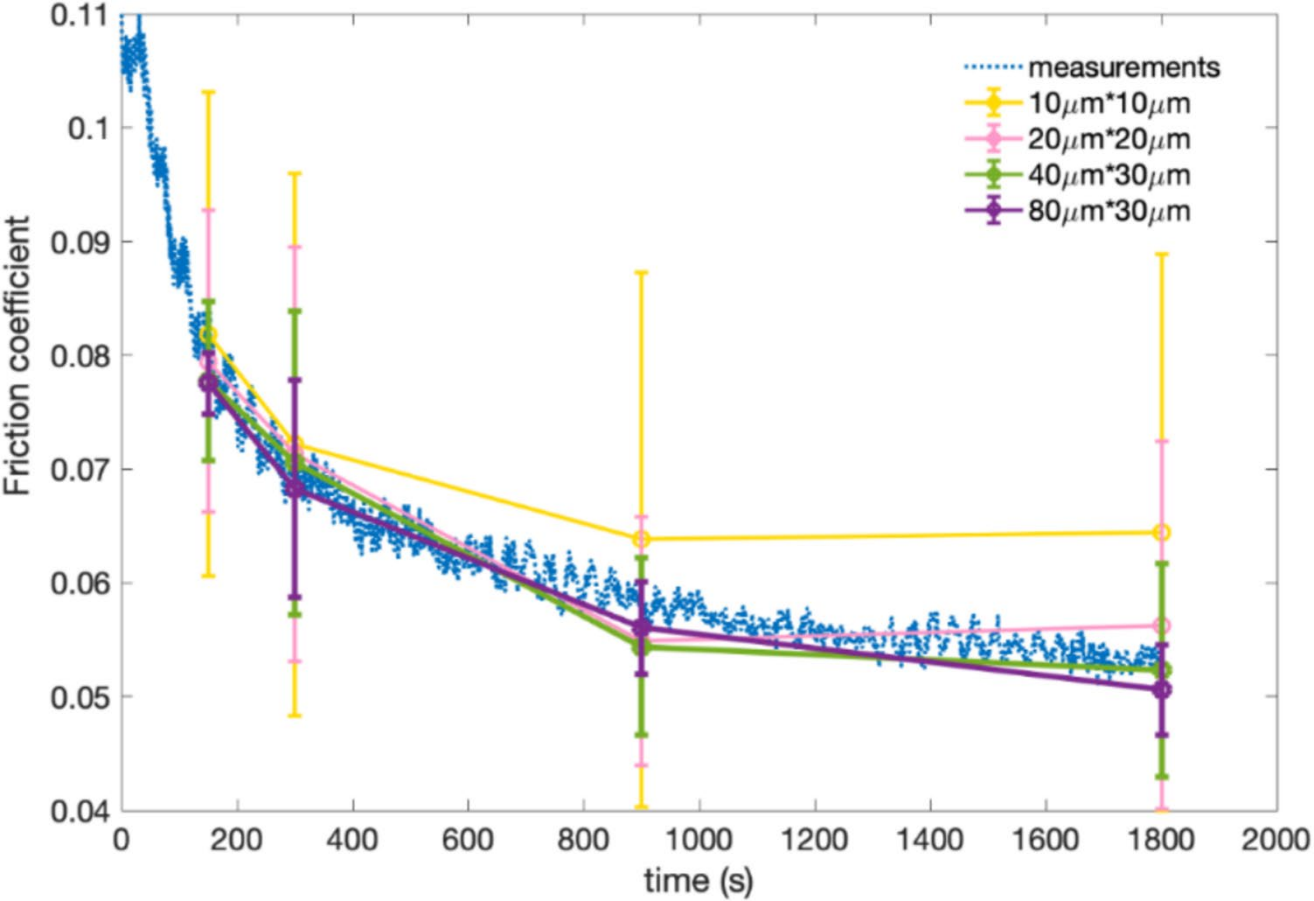
Friction coefficient as rubbing time, calibrated from Raman maps with different dimensions, compared to the measurements

To demonstrate the effect of Raman map size on the MoS_2_ quantitative analysis, a series of dimensions were collected from the same experimental data, and then the friction coefficients were determined from Raman intensity maps using a linear model assuming a direct relationship between the friction coefficient and the amount of MoS₂ tribofilm in the local tribocontact. This model was originally developed based on experimental data from a previous study [25] (Page 13,528). According to the model, the microscopic friction coefficient within the local tribocontact decreases linearly with increasing A₁_g_ peak intensity until a threshold value is reached, after which the friction reduction ceases. Comparisons between the measurements and values calibrated from different dimensions of Raman maps using linear model are shown in Fig. 3. The friction coefficients calibrated from micro-scale Raman maps deviate from the measured values, while the friction coefficients from the macro-scale Raman maps of 80 × 30 μm^2^ can best fit the curves of the measurements. Accordingly, Table 1 also shows that friction coefficients calibrated from macro-scale Raman maps of 80 × 30 μm^2^ are in accordance with the measurements, while low goodness of fit has been presented for other dimension of Raman maps.

**Table 1:** Goodness of fit between friction coefficient calibrations for different dimension of Raman maps and measured values

Dimension (μm^2^)	10 × 10	20 × 20	40 × 30	80 × 30
*R*^2^	0.4999	0.8672	0.8795	0.9255

**Fig. 4:**
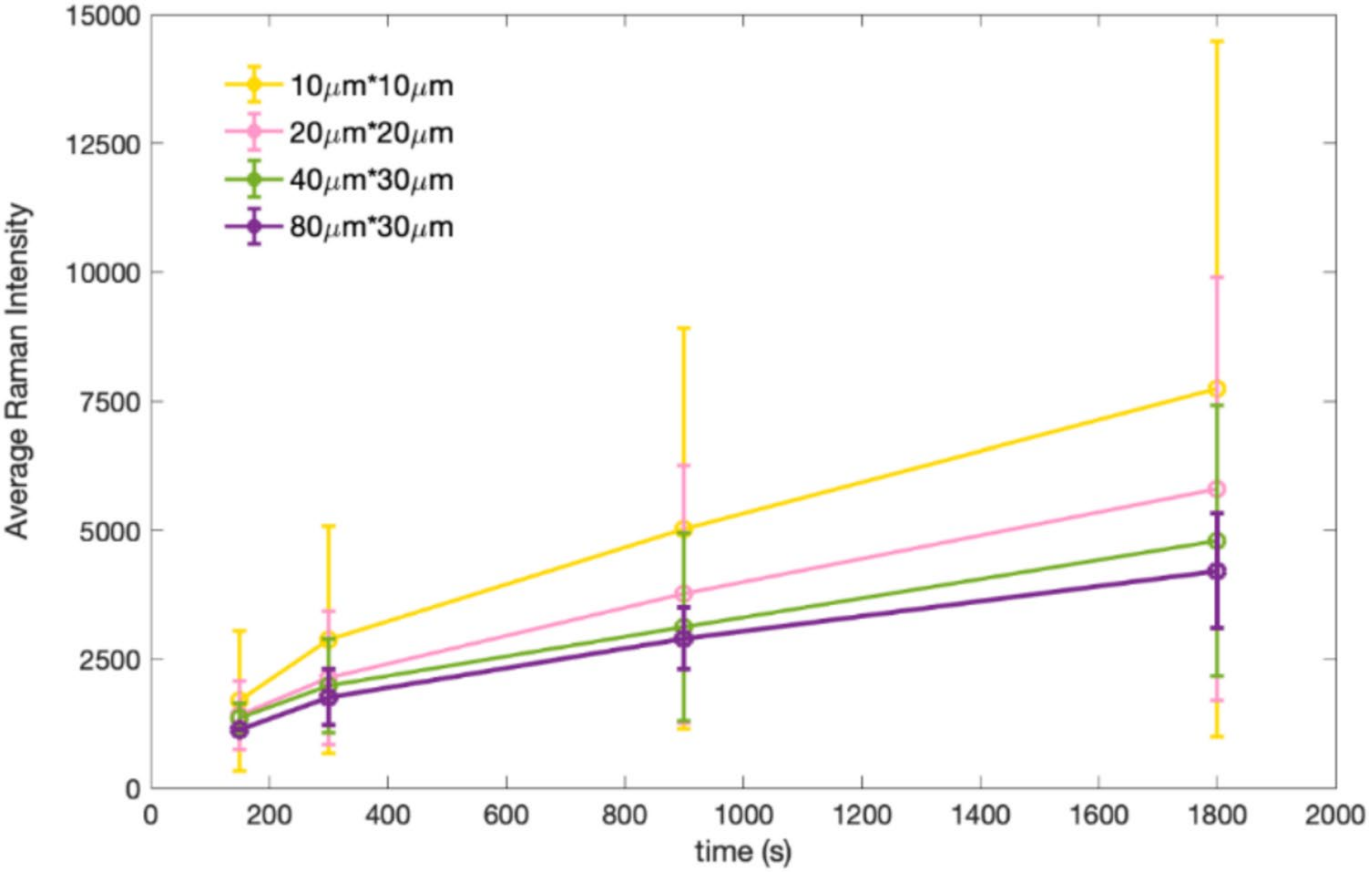
Average Raman intensity as rubbing time with different dimensions used to collect the Raman mapping data measurements

Then, the average A_1g_ peak intensity was calculated for each map, as plotted in Fig. 4. From Fig. 4, it can be suggested that the average MoS_2_ concentration increases with rubbing time, given that higher A_1g_ peak intensity means higher MoS_2_ concentration. In addition, the variations in the average Raman intensity in microscopic Raman maps (such as the Raman map of 10 × 10 μm^2^) are much larger than those of larger areas. Apparently, only a little difference can be seen in the average Raman intensity of the macro-scale Raman maps of 80 × 30μm^2^ at each rubbing time.

Table 2 further demonstrates that the values of average Raman intensity decrease as the dimension of the Raman map increases for each rubbing time. The mean value represents the average Raman intensity of the A₁_g_ peak, characteristic of MoS₂. It is calculated by summing the individual A₁_g_ peak intensities from all measured spectra and dividing by the total number of spectra. This parameter serves as a direct measure of the overall MoS₂ signal within the analyzed area. Additionally, r.m.s sampling errors were significantly reduced when macroscopic Raman maps were collected. It can be suggested that the micro-scale Raman maps with unacceptably high r.m.s sampling errors cannot be good representatives of MoS_2_ tribofilm concentration and distribution, such as the Raman map of 10 × 10 μm^2^ with the r.m.s error of 0.90. In contrast, the macroscopic Raman maps of 80 × 30 μm^2^ (data from four maps for each specimen) gave very low r.m.s sampling errors, which statistically verifies that four maps with this dimension are sufficient to achieve an acceptable calibration (r.m.s error is no more than 0.17).

**Table 2:** Data from Raman map calibrations with different dimensions

	2.5 min		5 min		15 min		30 min	
Dimension (μm^2^)	Mean	r.m.s error	Mean	r.m.s error	Mean	r.m.s error	Mean	r.m.s error
10 × 10	1688.72	0.64	2869.21	0.73	5029.45	0.67	7733.24	0.90
20 × 20	1415.81	0.32	2141.05	0.48	3757.65	0.44	5802.55	0.60
40 × 30	1356.35	0.21	1984.60	0.40	3119.80	0.36	4798.30	0.44
80 × 30	1187.54	0.07	1635.90	0.17	2723.12	0.17	3530.33	0.13

### Effect of Grid Density

In general, it would be expected that using denser grids will present more details in the Raman map, but it should also be noticed that the spatial resolution for Raman spectroscopy used here is around 1 μm. So, the minimum interval between two laser spots must be larger than this limitation in case the neighbouring laser spots overlap. The grid points are 2400 for each Raman map of 80 × 30 μm^2^ with 1 μm interval, which results in more than 13 h of exposure time for each measurement (considering 20 s exposure time for one single laser excitation). This would significantly increase the risk of damaging the tribofilm surface under a long-time of laser excitation. Thus, the minimum interval applied here is 2 μm in the X and Y directions.

**Fig. 5:**
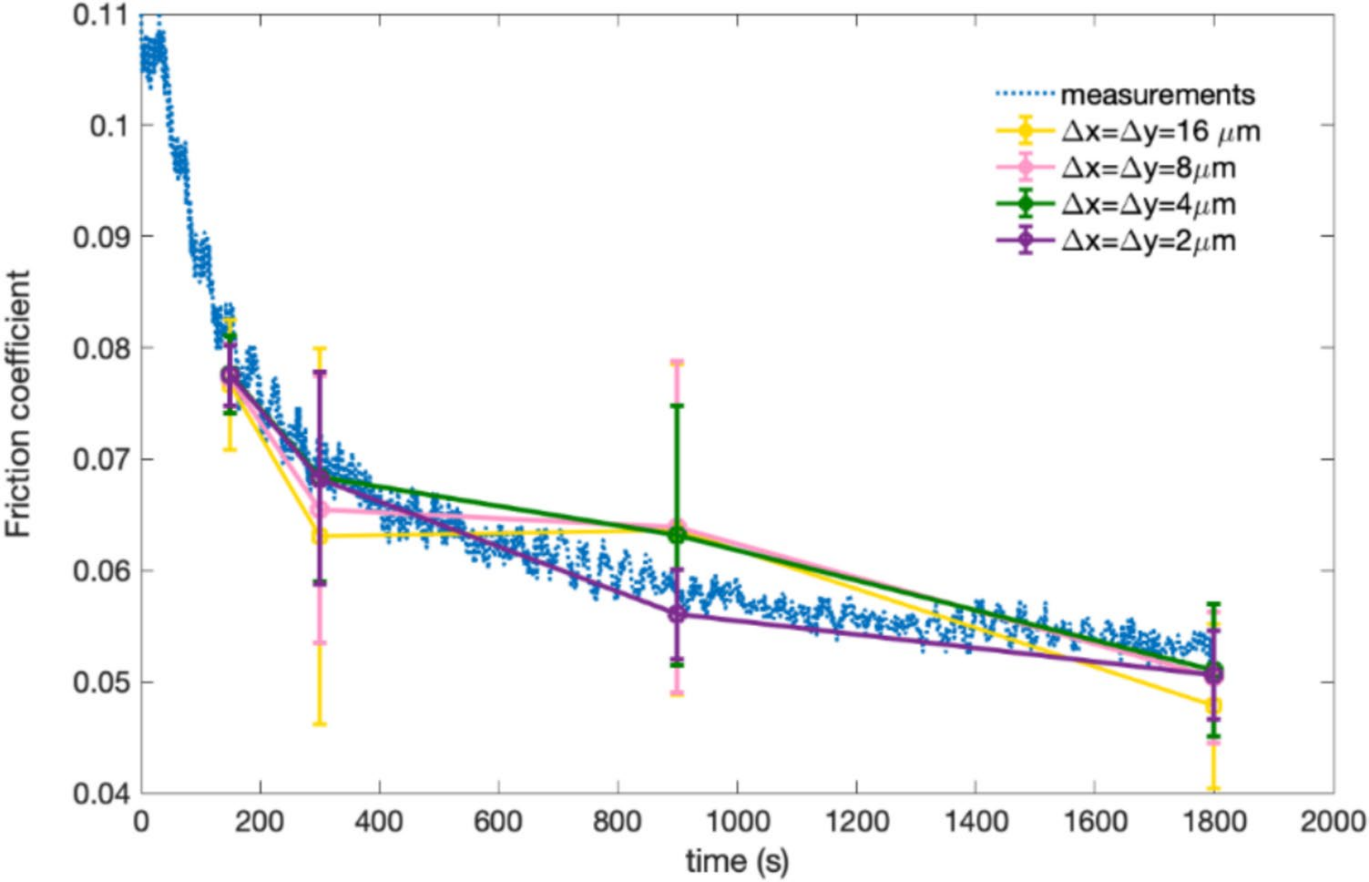
Friction coefficient as a function of sliding time with measured values and calibrations from Raman maps with different mesh grids

Figure 5 shows the comparisons between the measured friction coefficients and those calibrated from Raman maps with different mesh grids. The predicted friction coefficients from Raman maps with 2 μm intervals can best fit the measured values. Table 3 compares the calibration results of the four Raman maps of 80 × 30 μm^2^ with different grid densities at each rubbing time. It can be seen in Table 3 that coarse grids may not affect the average Raman intensity results but will result in high sampling errors compared to the calibrations with 2 μm intervals.

**Table 3:** Data from raman map calibrations with different grid density

Intervals (μm)	*R*^2^	2.5 min		5 min		15 min		30 min	
		Mean	r.m.s error	Mean	r.m.s error	Mean	r.m.s error	Mean	r.m.s error
2 × 2	0.93	1187.5	0.07	1635.9	0.17	2723.1	0.17	3530.3	0.13
4 × 4	0.92	1431.0	0.37	1641.4	0.27	2647.0	0.25	3727.9	0.24
8 × 8	0.88	1416.1	0.26	1611.7	0.38	2680.0	0.38	3889.2	0.35

## Numerical Model

**Fig. 6:**
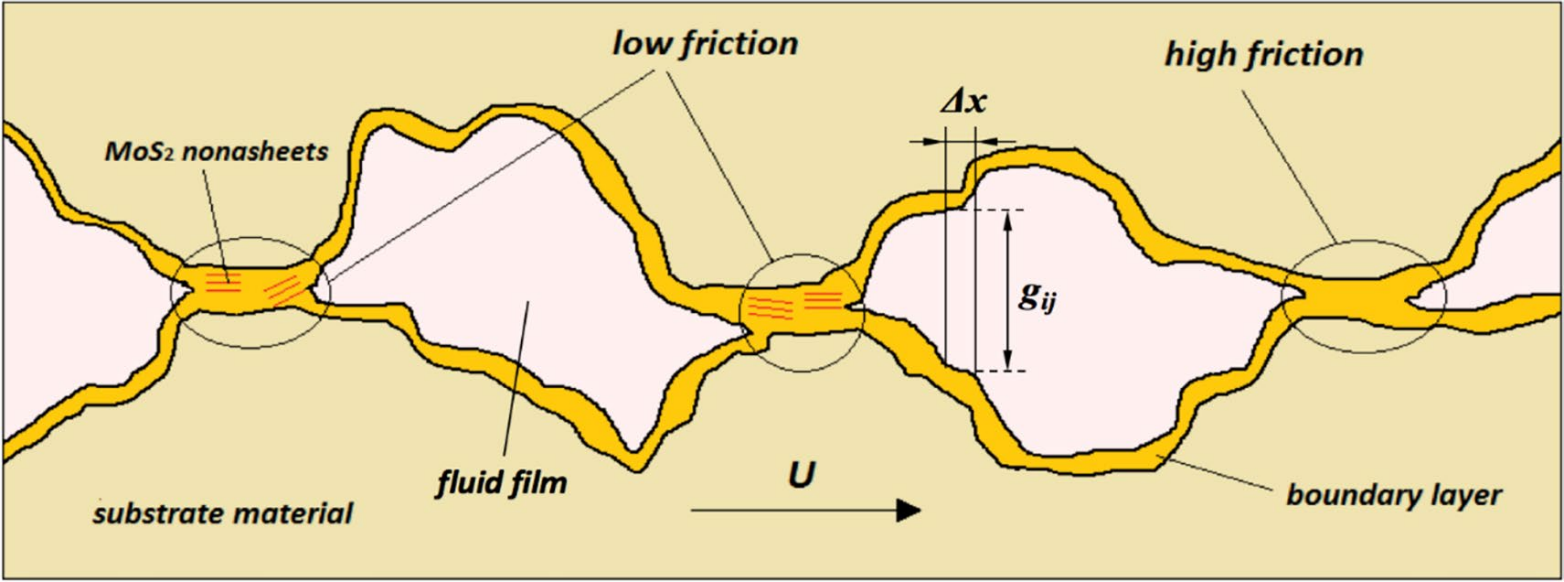
Illustration of boundary lubricated surfaces with MoDTC/ZDDP tribofilm

The low-shear-strength MoS_2_ nanocrystal sheets are formed on MoDTC/ZDDP tribofilms due to MoDTC additive decomposition. The present model monitors the concentration of MoS_2_ in the tribofilm, linking it to friction reduction. To simplify the model, the following assumptions are made in this study:

- Only the pressures applied on the contacted asperities are considered in the boundary lubrication model. The base fluid of the lubricant behaves like a transfer media of additives, and hydrodynamic effects are neglected in the present model.
- The shear stress from the confined lubricant in the micro valleys is transferred to the MoDTC molecules between the surfaces and activates the decomposition of MoDTC [15].
- A tribo-activated Arrhenius-type equation was employed to capture the tribofilm growth due to the stress-augmented thermal activation [20].
- In the previous study of the authors [25](Page 13,528), once the local amount of MoS_2_ tribofilm exceeds the pre-set threshold in the contacted area, a constant low friction coefficient value is obtained within this region, regardless of the crystallinity and orientation of MoS_2_ flakes in the tribofilm matrix.
- The formation of the MoS_2_ tribofilm is activated by the tribochemical reaction, while the removal is a physical exfoliation of MoS_2_ from the MoDTC/ZDDP tribofilm matrix.
- Other compounds in the tribofilm matrix (like oxides, polyphosphates, etc.) are regarded as a one-layer system (“boundary layer” as shown in Fig. 6) evenly distributed between the rubbing surfaces.

### MoS_2_ Tribofilm Formation

In stress-augmented thermal activation, localised stress decreases the activation energy barrier, exponentially accelerating the rate of atomic and molecular processes. According to stress-augmented thermal activation, the formation rate of MoS_2_ tribofilm can be expressed by a tribo-activated Arrhenius model [11, 20],

1 $$\left(\frac{{{\text{d}} C}}{{{\text{d}} t}} \right)_{{\text{formation}}} = k_{{\text{tribo}}} \cdot A$$

and

2 $$k_{{\text{tribo}}} = \chi \cdot k_{thermal} (T)$$

**Fig. 7:**
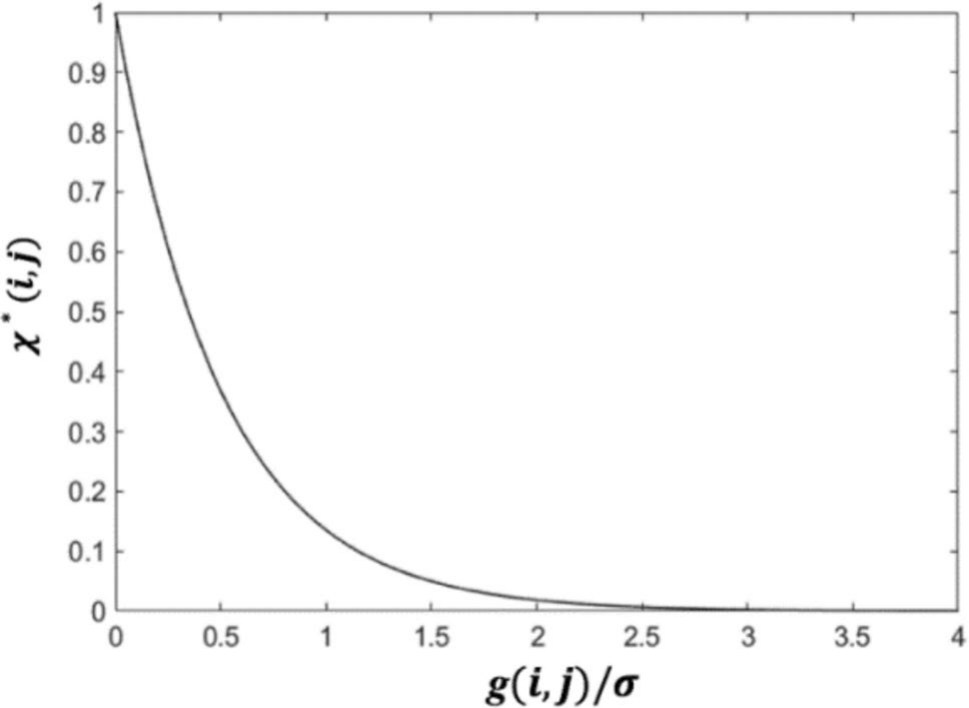
Illustration of the relationship in Eq. (4), where *b*_1_ = 2

*A* is the concentration of the MoDTC additive in the lubricants. This Arrhenius-type equation models the thermal growth of the tribofilm, incorporating a multiplication of the rate coefficient by a parameter, *χ*, to account for the enhanced growth rate due to mechanoactivation. The reaction rate of the thermal activation *k*_thermal_ depends on the surface temperature (sum of the base oil temperature and the flash temperature at tribocontacts), and thus, based on the Arrhenius equation for a thermoactivated reaction, *k*_thermal_ can be expressed by

3 $$k_{thermal} (T) = C_{1} \cdot \exp \left( - \frac{{C_{2} }}{T} \right),$$

where *C*_1_ is the pre-factor, and the constant *C*_2_ consists of the internal activation energy for the MoS_2_ formation as well as the Boltzmann’s constant. This tribo-factor *χ* is related to the contact severity, which is determined by the surface roughness, contact pressure, and the surface hardness [30, 31], as shown in the following equation [10],

4 $$\chi (i,j) = b_{0} \exp \left( { - b_{1} \cdot \frac{g(i,j)}{{\sigma^{*} }}} \right),$$

where *b*_0_, *b*_1_ are constants, *g*(*i*,*j*) represents the gap between the rough surfaces at the mesh grid (*i*,*j*) as shown in Fig. 1, σ* denotes the composite roughness and ${\sigma }^{*}=\sqrt{{R}_{{q}_{1}}^{2}+{R}_{{q}_{2}}^{2}}$. Based on Eq. (4), the dimensionless χ* (χ* = $\chi /{b}_{0}$) can be visualised in Fig. 7.

Thus, the MoS_2_ tribofilm formation rate can be given by

5 $$\left(\frac{{{\text{d}} C}}{{{\text{d}} t}} \right)_{{\text{formation}}} = \chi \cdot C_{1} \cdot \exp \left( - \frac{{C_{2} }}{T} \right) \cdot A$$

Combining with the Eq. (4), then

6 $$(\frac{{{\text{d}} C}}{{{\text{d}} t}})_{{\text{formation}}} = C_{1}{\prime} \exp \left( { - b_{1} \cdot \frac{g(i,j)}{{\sigma^{*} }}} \right) \cdot \exp ( - \frac{{C_{2} }}{T}),$$

where $${C}_{1}{\prime}={b}_{0}{C}_{1}A$$.

### *Combined MoS*_*2*_* Formation and Removal*

The dynamic growth of MoS_2_ is controlled by two antagonistic processes: the formation and removal of MoS_2_, which can be expressed as follows,

7 $$\left(\frac{{{\text{d}} C}}{{{\text{d}} t}} \right)_{{\text{growth}}} = \left(\frac{{{\text{d}} C}}{{{\text{d}} t}} \right)_{{\text{formation}}} + \left(\frac{{{\text{d}} C}}{{{\text{d}} t}} \right)_{remove}$$

With mathematical simplification and assuming a linear relationship between the Raman intensity and the amount of crystalline MoS_2_ tribofilm in the MoDTC/ZDDP tribofilm matrix [32],

8 $$I = k_{0} \cdot C$$

and then

9 $$\left(\frac{{{\text{d}} I}}{{{\text{d}} t}} \right) = k_{0} \cdot \left(\frac{{{\text{d}} C}}{{{\text{d}} t}} \right)$$

The Eq. (7) can be substituted as

10 $$(\frac{{{\text{d}} I}}{{{\text{d}} t}})_{{\text{growth}}} = (\frac{{{\text{d}} I}}{{{\text{d}} t}})_{{\text{formation}}} + (\frac{{{\text{d}} I}}{{{\text{d}} t}})_{remove}$$

*In-situ* Raman spectroscopy was employed to monitor the removal of MoS₂ tribofilms as a function of rubbing time. Initially, the MoDTC/ZDDP tribofilm was formed over 1 h using a base oil containing 1 wt% ZDDP and 0.5 wt% MoDTC. Subsequently, the tribometer was halted to drain the initial oil and replace it with fresh oil containing only 1 wt% ZDDP (no MoDTC additive), thereby initiating the tribofilm removal process.

During the removal phase, Raman spectra were periodically collected from the same location on the disc wear scar using a 488 nm laser excitation source at 50% laser power and a 20-s exposure time. To enhance measurement accuracy and reduce positional and resolution errors inherent in in-situ Raman analyses, a 3 × 3 μm^2^ Raman map was conducted, resulting in nine individual Raman spectra. The average normalized intensity of the A₁_g_ peak was calculated from these spectra, serving as a quantitative measure of the MoS₂ tribofilm amount. The decrease in normalized A₁_g_ peak intensity over time was modeled using the following equation:

11 $$\left( {\frac{dI}{{dt}}} \right)_{remove} = - C_{4} \left( {1 - e^{{ - C_{5} t}} } \right)$$

which facilitates the quantification of tribofilm removal dynamics, aligning with the methodological framework established in our previous study [25].

In this work we have chosen the growth of the A_1g_ Raman peak for MoS_2_ to be the quantitative measure for the growth of the tribofilm. Therefore, combining the Eqs. (4), (6), (11), the growth of the A_1g_ peak intensity at spot (*i*,* j*) within the tribocontact areas can be given by the following equation,

12 $$I_{n + 1} (i,j) = I_{n} (i,j) + \Delta t \cdot \left[ {C_{3} \cdot \exp \left( { - b \cdot \frac{g(i,j)}{{\sigma^{*} }}} \right) \cdot \exp ( - \frac{{C_{2} }}{T(i,j)}) - C_{4} \cdot (1 - e^{{ - C_{5} t}} )} \right],$$

where *C*_3_ = *k*_0_·*C*_1_· *b*_0_· *A*.

## Full Numerical Simulation

**Fig. 8:**
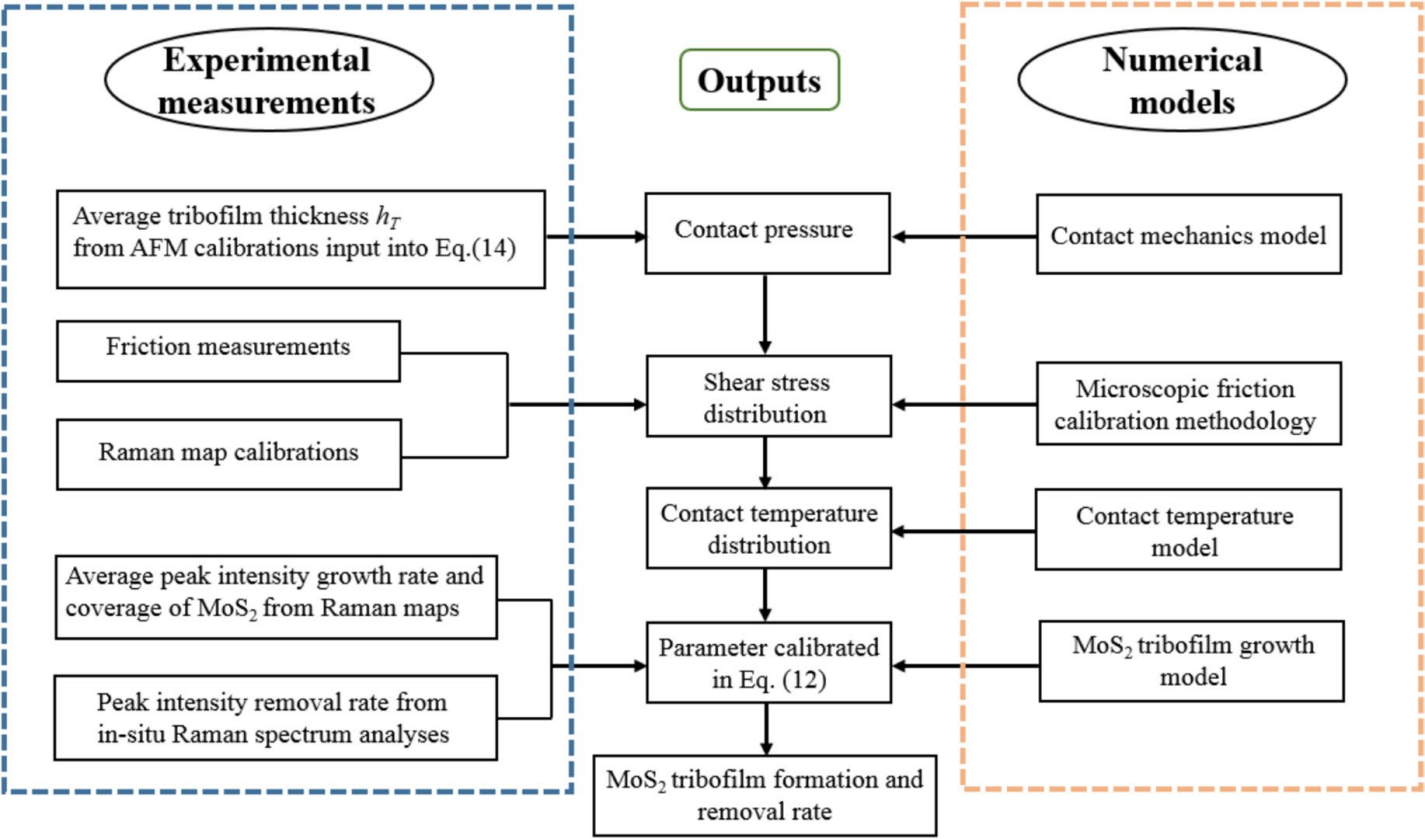
Illustration of the calibration procedure on the experimental works coupled with numerical models, to output the MoS_2_ tribofilm formation and removal rate

A specific calibration procedure will be carried out in the following section, to yield the constants in terms of the MoS_2_ growth dynamics in Eq. (12). Figure 8 illustrates how the experimental measurements and the numerical modelling have been coupled in the calibration procedure. Subsequently, these calibrated parameter values will be used in the full numerical simulations to predict the quantity of MoS_2_ tribofilm on the rubbing surfaces and resultant friction coefficient.

### Calibration

The tribotests were conducted using a ball-on-disc tribometer under boundary lubricated conditions. Consequently, the simulation models the contact between a flat surface and a sphere, with the steel ball against the rotating disc. The steel discs featured inner and outer diameters of 85 mm and 110 mm, respectively, while the ball bearing had a diameter of 6.35 mm. The geometry of the surface profiles can be inferred from these dimensions. Table 4 lists the surface materials, lubricants, and test conditions from the tribotests.

**Table 4:** Conditions for tribological tests in the boundary lubrication regime

Samples	Disc: AISI 1074, ball: AISI 52100
Dimensions	Inner/outer diameter of the disc: 85 mm/110 mm Diameter of the ball: 6.35 mm
Roughness r.m.s	Disc: 200 nm, ball:17 nm
Young’s modulus	210 GPa (Disc and Ball)
Poisson ratio	0.3 (Disc and Ball)
Absolute Viscosity (mPa⋅s)	10.5 (80 °C)
Pressure-viscosity coefficient (GPa^−1^)	12.1 (80 °C)
Lambda ratio	≤ 0.038
Density of the solids, *ρ*_s,1/2_ (kg/m^3^)	7800.0
Specific heat of the solids, *c*_s,1/2_ (J/kg K)	460.0
Solids thermal diffusivity, *α*_s_ (mm^2^/s)	13.378

AFM analysis showed neighboring asperities on the tribofilm-coated discs were about 2 µm apart across the wear scar [25](Page 13,526). This justified setting up 64 × 64 mesh grids to accurately map a rough surface area of 90 × 90 µm^2^. The surface roughness was digitally generated and superposed on the smooth surface profiles of the ball and disc, respectively. The dimensionless size of the calculation grid corresponds to a square area of -1.5 $\le$ X $\le$ 1.5 and -1.5 $\le$ Y $\le$ 1.5.

#### Contact Mechanics Model

In this model, the local gap and the contact pressure are calculated from the contact mechanics model considering elastic/fully plastic deformation. The obtained contact pressure is then an input for calculating the flash temperature. The elastic deformation (*u*_e_) caused by both normal contact pressures (*p*_a_) and tangential pressures (*τ*_*s*_) can be calculated using the Boussinesq and Cerrutti equations.

13 $$u_{e} (x,y) = \frac{2}{{\pi E^{*} }}\iint\limits_{\Omega } {\frac{{p_{a} (s,t)}}{{\sqrt {(x - s)^{2} + (y - t)^{2} } }}}dsdt - \frac{1}{\pi G}\iint\limits_{\Omega } {\frac{{\tau_{a} (s,t) \cdot (s - x)}}{{(x - s)^{2} + (y - t)^{2} }}}dsdt,$$

where $$E^{*} = {1 \mathord{\left/ {\vphantom {1 {\left[ {\frac{1}{2}(\frac{{1 - \upsilon_{1}^{2} }}{{E_{1} }} + \frac{{1 - \upsilon_{2}^{2} }}{{E_{2} }})} \right]}}} \right. \kern-0pt} {\left[ {\frac{1}{2}(\frac{{1 - \upsilon_{1}^{2} }}{{E_{1} }} + \frac{{1 - \upsilon_{2}^{2} }}{{E_{2} }})} \right]}}$$, and $$\frac{1}{G} = \frac{1}{2}\left[ {\frac{{(1 + \upsilon_{1} )(1 - 2\upsilon_{1} )}}{{E_{1} }} - \frac{{(1 + \upsilon_{2} )(1 - 2\upsilon_{2} )}}{{E_{2} }}} \right]$$.

The Fast Fourier Transform (FFT) method (based on the work of Liu et al. [33] and Wang et al. [34]) is used to calculate the elastic deformation. Then the numerical algorithm based on the conjugate gradient method (CGM) is implemented to determine the contact pressure within the real surface areas [35, 36].

Once elastic deformations, as well as the contact pressures are determined, a plastic deformation model proposed by Almqvist et al. [37] is executed to acquire the permanent deformations at the tribocontacts. The tribofilm has gradient features in the mechanical properties, and the threshold of contact pressure obeys the following boundary conditions, the same as those used in the work of Andersson et al*.* [30],

14 $$p_{{threshhold}} = \left\{ \begin{gathered} \begin{array}{*{20}c} {H_{s} - \frac{{H_{s} - H_{T} }}{{h_{T} }}(h_{T} - u_{p} ),} & {u_{p} < h_{T} } \\ \end{array} \hfill \\ \begin{array}{*{20}c} {} & {} & & {\begin{array}{*{20}c} {H_{s} } & {} & {\begin{array}{*{20}c} {} & , & {u_{p} \ge h_{T} } & {} \\ \end{array} } & {} \\ \end{array} } \\ \end{array} \hfill \\ \end{gathered} \right.$$

where *h*_T_ is the average MoDTC/ZDDP tribofilm thickness. This means that the tribofilm would bear the load if the plastic deformation is no more than the tribofilm thickness, and the pressure threshold follows a linear relation with the plastic deformation until the plastic penetration reaches the substrate. The average tribofilm thickness as a function of time was measured by the post-test AFM calibration (experimental data available in Page 13,530 of Ref. [25]).

#### Microscopic Friction Calibration Methodology

The microscopic friction coefficients at tribocontacts were then obtained using the Raman peak intensity maps, assuming a linear relationship between the microscopic friction and the local amount of MoS_2_ tribofilm [25](Page 13,528),

15 $$\mu (i,j) = \left\{ \begin{gathered} \mu _{{{\text{high}}}} - \frac{{\mu _{{{\text{high}}}} - \mu _{{{\text{low}}}} }}{{I_{{{\text{threshold}}}} }} \times I(i,j),\begin{array}{*{20}c} {I(i,j) < I_{{{\text{threshold}}}} } & {} \\ \end{array} \hfill \\ \mu _{{{\text{low}}}} ,\begin{array}{*{20}c} {\begin{array}{*{20}c} {\begin{array}{*{20}c} {} & {} \\ \end{array} } & {} & {} & {} \\ \end{array} } & {} & {I(i,j) \ge I_{{{\text{threshold}}}} } & {} \\ \end{array} \hfill \\ \end{gathered} \right.$$

where *μ*(i,j) and *I*(i,j) denote the microscopic friction coefficient and local Raman peak intensity at the spot (i, j). The microscopic friction specifically denotes the frictional behavior occurring at the scale of individual asperity contacts within the local tribocontact area. Then, the macroscopic friction coefficient, which refers to the bulk frictional properties within the wear track, can be determined by averaging the microscopic friction coefficients obtained from Eq. 15. The coverage of the MoS_2_ tribofilm can be quantified using Raman intensity maps by applying a specific threshold of 2295 counts for the A₁_g_ peak intensity, determined based on previous experimental calibration [25](Page 13,529). By mapping areas exceeding this threshold, we obtained a quantitative measure of MoS₂ coverage, ensuring accurate differentiation between covered and uncovered areas. Once the microscopic friction coefficient is determined, the shear stress distribution within the contact area could be acquired immediately and then used to calculate the contact temperature on the rubbing surface.

#### Contact Temperature Model

The heat flux generated at the interface of tribocontacts can be written as,

16 $$q_{i,j} = \mu_{i,j} \cdot p_{i,j} \cdot u_{s}$$

The surface temperature rise at any point (*x*,*y*) on either of the two surfaces at any time *t* due to a transient heat source of strength *q*(*x*’,*y*’,0,*t*’) d*x*’d*y*’d*t*’ at the point (*x*’,*y*’) at time *t*’ obeys the following equation given by Carslaw and Jaeger [38],

17 $${\text{d}} T(x,y) = \frac{{q(x^{\prime},y^{\prime},0,t^{\prime}){\text{d}} x^{\prime}{\text{d}} y^{\prime}{\text{d}} t^{\prime}}}{{4\rho {}_{s}c{}_{s}[\pi \alpha {}_{s}(t - t^{\prime})]^{3/2} }}\exp \{ - \frac{{[(x - x^{\prime}) - u(t - t^{\prime})]^{2} + (y - y^{\prime})^{2} }}{{4\alpha {}_{s}(t - t^{\prime})}}\}$$

with *ρ*_s_ is the density of the solids, *c*_s_ denotes the specific heat of the solids, and *α*_s_ denotes the thermal diffusivity of the solid. In this work, the Fast Fourier Transform (FFT) is used to speed up the discrete convolution of flash temperature calculation. More details on the numerical solution to flash temperature can be found in the work of Zhao et al. [39]. Finally, the surface temperature at the tribocontact on surfaces 1 and 2 can be expressed by the following equation,

18 $$\begin{array}{*{20}c} {T_{r} = T_{oil} + \Delta T_{r} ,} & {r = 1,2} \\ \end{array}$$

By then, the surface gaps and contact temperatures have been determined from experimental measurements coupled with the numerical models, and subsequently were input in Eq. (12).

#### *MoS*_*2*_* Tribofilm Growth Model*

**Fig. 9:**
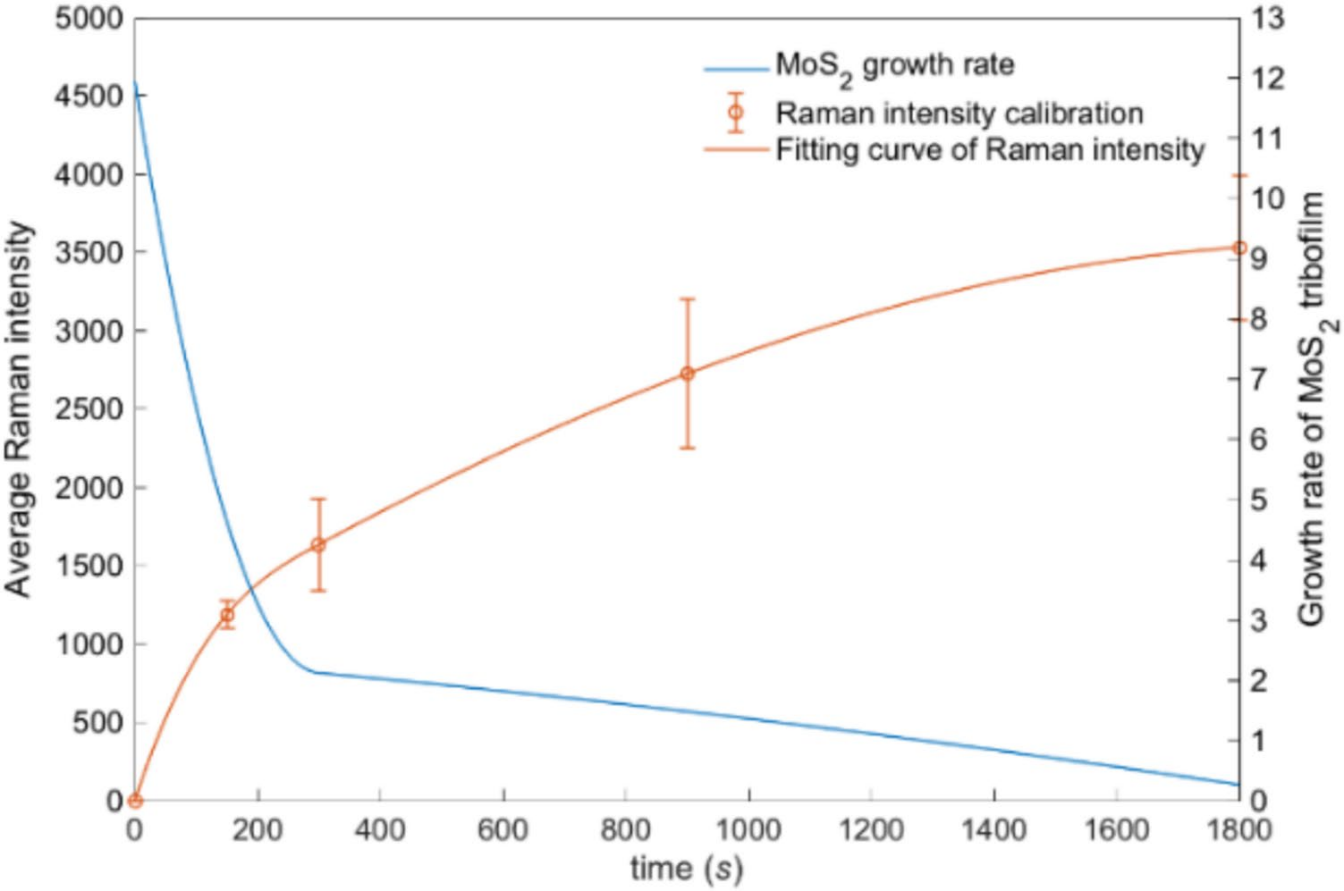
Raman map calibration and corresponding growth rate of MoS_2_ tribofilm at 80 °C

The left hand of Eq. (12) is the growth rate of A_1g_ peak Raman intensity. The average A_1g_ Raman intensity, as plotted in Fig. 9, can be calibrated from the measured time-resolving Raman intensity maps (see Sect. 2). Then, the growth rate of MoS_2_ (denoted as the growth rate of peak intensity in Fig. 9) can be obtained by differentiating the average Raman intensity with respect to time. It is worth noticing that the growth rate drops rapidly with sliding, and after around 300 s of sliding, it levels out at a steady-state.

The formation of MoS₂ tribofilms theoretically ceases when the surface is entirely covered, corresponding to a coverage parameter *α* = 1. This termination occurs because the adsorption of MoDTC molecules becomes increasingly constrained as MoS₂ coverage grows, thereby reducing the available nascent areas for further adsorption. However, the experimental results consistently indicate coverage values below unity [25](Page 13,532). This limitation arises from incomplete surface contact and the dynamic removal of MoS₂ nanosheets during testing, which impede the attainment of full tribofilm coverage. Additionally, the extent of MoS₂ coverage influences its removal rates; regions with greater tribofilm coverage exhibit enhanced removal rates, facilitating more efficient detachment during subsequent tribological cycles. Therefore, the average growth rate of MoS_2_ can be expressed by the following equation,

19 $$\left( {\frac{{dI_{m} }}{dt}} \right)_{growth} = (1 - \alpha^{a} ) \cdot \left( {\frac{{dI_{m} }}{dt}} \right)_{formation} - \alpha^{4} \cdot C_{4} \left( {1 - e^{{ - C_{5} t}} } \right)$$

**Fig. 10:**
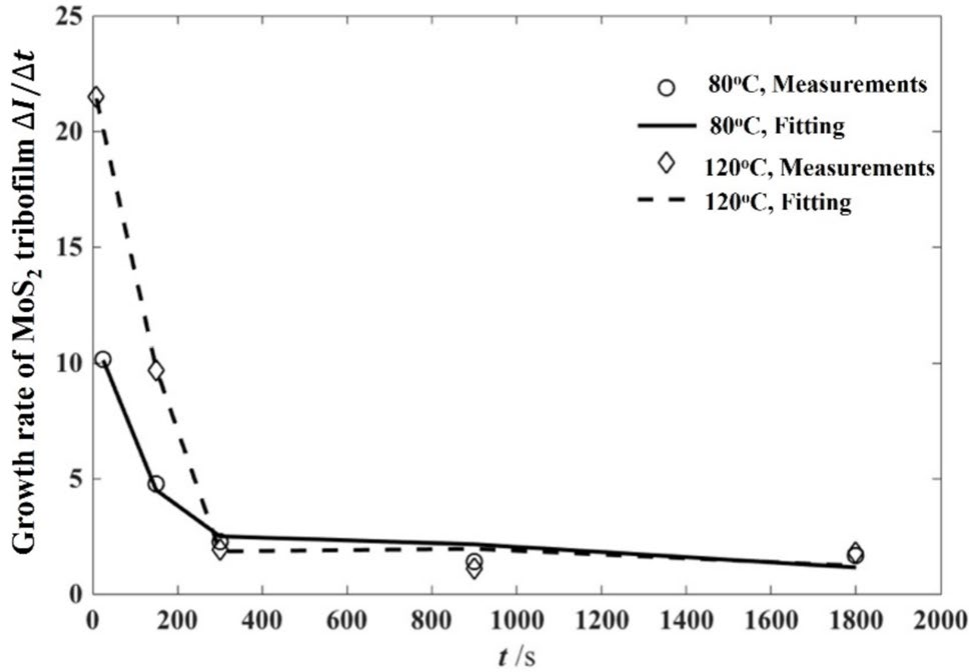
Calibration results of the average growth rate of MoS_2_ tribofilm at 80 and 120 °C

The *in-situ* Raman spectroscopy was employed to monitor the removal of MoS_2_ from tribofilms at different temperatures. The removal rates denoted as the constant *C*_5_ in Eq. (12) were found from the fitted curves for the normalised intensity of the A_1g_ peak removal, with the values at different temperatures shown in Table 5. Finally, the constants in terms of MoS_2_ tribochemical dynamics in Eq. (12) can be found from the fitted curves demonstrated as solid lines in Fig. 10, with the best goodness of fit achieved. The constant values for the 80 and 120 °C conditions are listed in Table 6.

**Table 5:** Constants calibrated from fitting curves of MoS_2_ removal

Temperature (°C)	${C}_{4}/{I}_{0}$	${C}_{5}$
80	0.5632	0.0050
120	0.8792	0.0064

**Table 6:** Parameters calibrated from the experimental results and used in the numerical simulations

Parameter	Value			Description
*T*_oil_	80 °C		120 °C	Test temperatures
*C*_3_	147.83			Pre-factor for the tribochemical reaction rate in Eq. (12)
*a*	0.4			Exponent for the coverage of MoS_2_
*C*_2_	468.1			Exponential constant for the thermochemical reaction rate in Eq. (12)
*C*_4_	1.91	3.07		Linear constant for the removal rate in Eq. (12)
*C*_5_ (s^−1^)	0.0050	0.0065		Exponential constant for the removal rate in Eq. (12)
*H*_steel_ (GPa)	5			Hardness of the substrate of steel
*H*_tribo_ (GPa)	2 GPa			Hardness of the MoDTC/ZDDP tribofilm matrix

### Model Validation

**Fig. 11:**
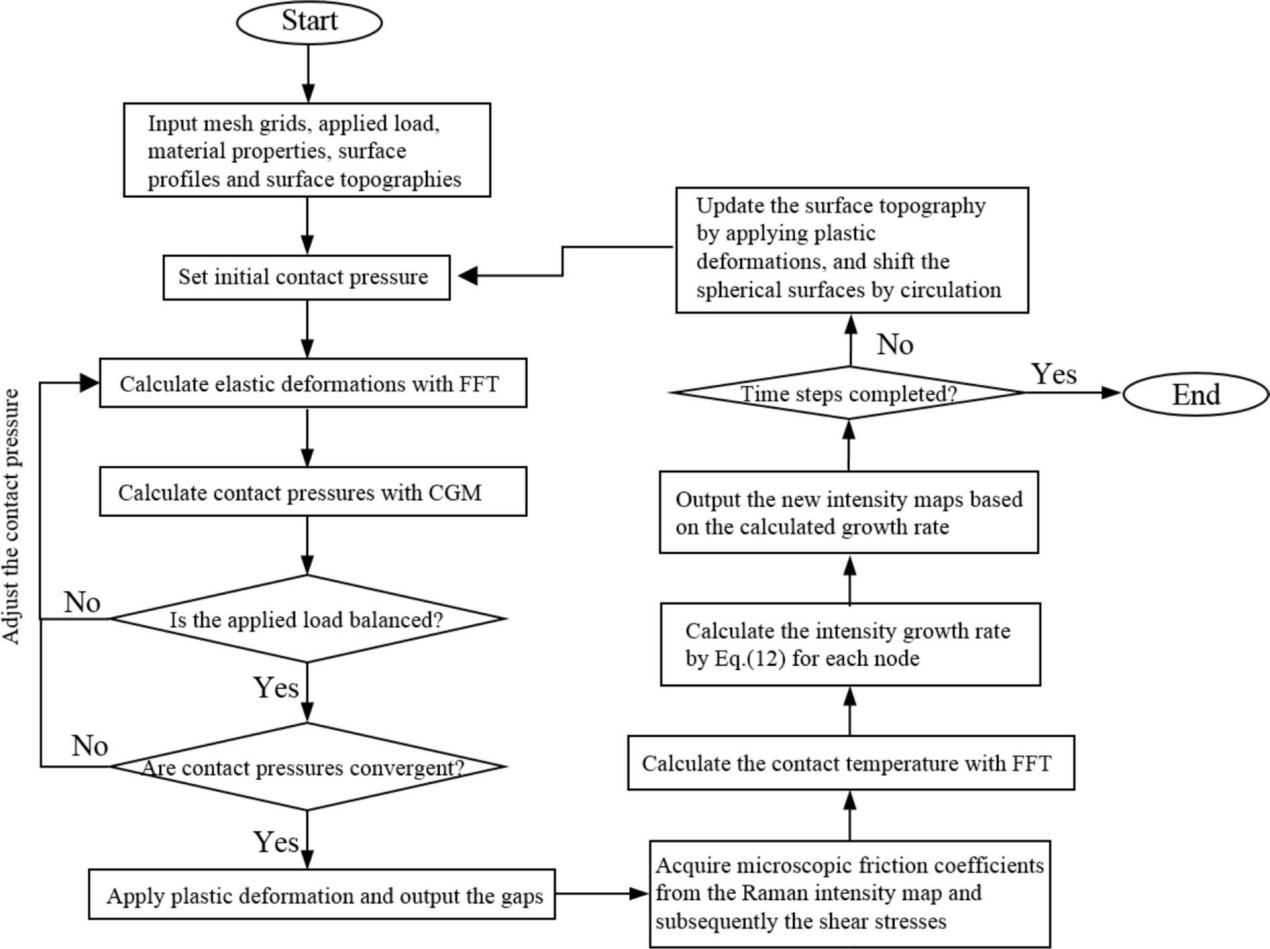
Flowchart illustrating the numerical procedure implemented in the MoS_2_ tribofilm growth model

Full numerical simulations were carried out using the resulting values on the calibrated parameters in Table 6, with regarding to rubbing under two rough surfaces at 80 and 120 °C oil temperatures in this part. Figure 11 illustrates the full numerical simulation procedure. To evaluate the simulation results, the mesh grids and dimension of the analyzed area used in the numerical simulations are the same as those presented in Sect. 4.1, and other parameters in terms of materials, lubricant properties, and test conditions employed in the simulations can be found in Table 4.

The Hertzian contact width is 45 µm with a sliding velocity of 0.255 m/s, which means the travel distance and sliding time for one contact calculation are 90 µm and 0.35 ms. In the contact temperature calculation loop, the time steps are chosen as *N*_t_ = 250, which means, in this case the time for one step is 1.4 µs. The time steps for the tribofilm growth are set up as *N* = 300, considering that the tribofilm growth occurs quite slowly and can keep the same value for a period of 6 s.

**Fig. 12:**
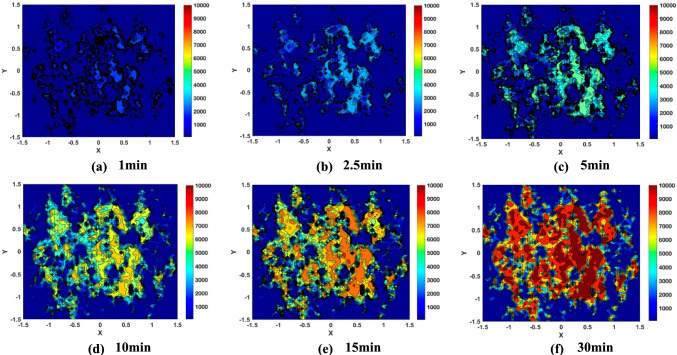
Simulated MoS_2_ (A_1g_ peak intensity) distributions in the centre of wear scar on the disc surface at 80 °C **a** 1 min **b** 2.5 min **c** 5 min **d** 10 min **e** 15 min **f** 30 min

**Fig. 13:**
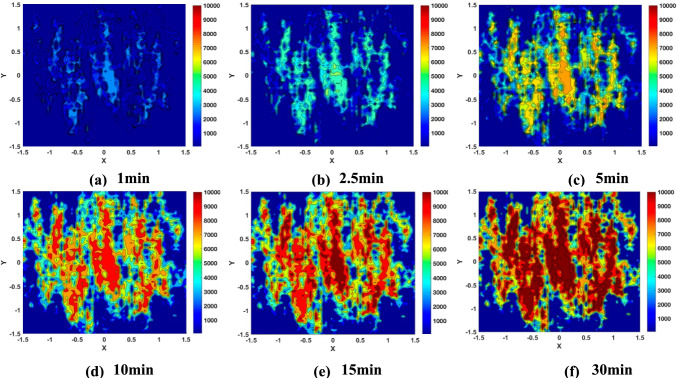
Simulated MoS_2_ (A_1g_ Peak Intensity) distributions in the centre of wear scar on the disc surface at 120 °C **a** 1 min **b** 2.5 min **c** 5 min **d** 10 min **e** 15 min **f** 30 min

Figures 12 and 13 present the simulation results of the A_1g_ peak intensity maps showing how the MoS_2_ tribofilm builds up on the disc surface as a function of rubbing time. These simulated Raman maps not only visualise how the MoS_2_ tribofilm distributions expand on the whole wear scar but also verify the local quantity accumulations of MoS_2_ tribofilm in the micro-scale during rubbing. The qualitative features of the simulated MoS₂ distributions—such as spatial patterns and coverage levels—demonstrate a strong correspondence with the experimental observations[25] (Figs S1 and S2) in Supplementary materials). The MoS_2_ tribofilms progressively expand in local areas of higher contact severity, where gaps between contacting surfaces are relatively smaller compared to surface roughness (see Fig. 7), at both temperatures. At 120 °C, regions exhibiting higher peak intensities are more widespread, suggesting greater coverage by the MoS_2_ tribofilms. These observations correspond with the measured Raman intensity maps. However, the simulated MoS₂ distributions were confined to the same localized contact area, providing a controlled environment for modelling tribofilm behaviour. In contrast, the experimental Raman maps were collected ex-situ from various regions within the wear track, reflecting a broader and more heterogeneous tribological environment. Directly matching simulated and experimental maps requires precise alignment of contact areas, which is challenging given the dynamic and variable nature of wear processes in experiments.

**Fig. 14:**
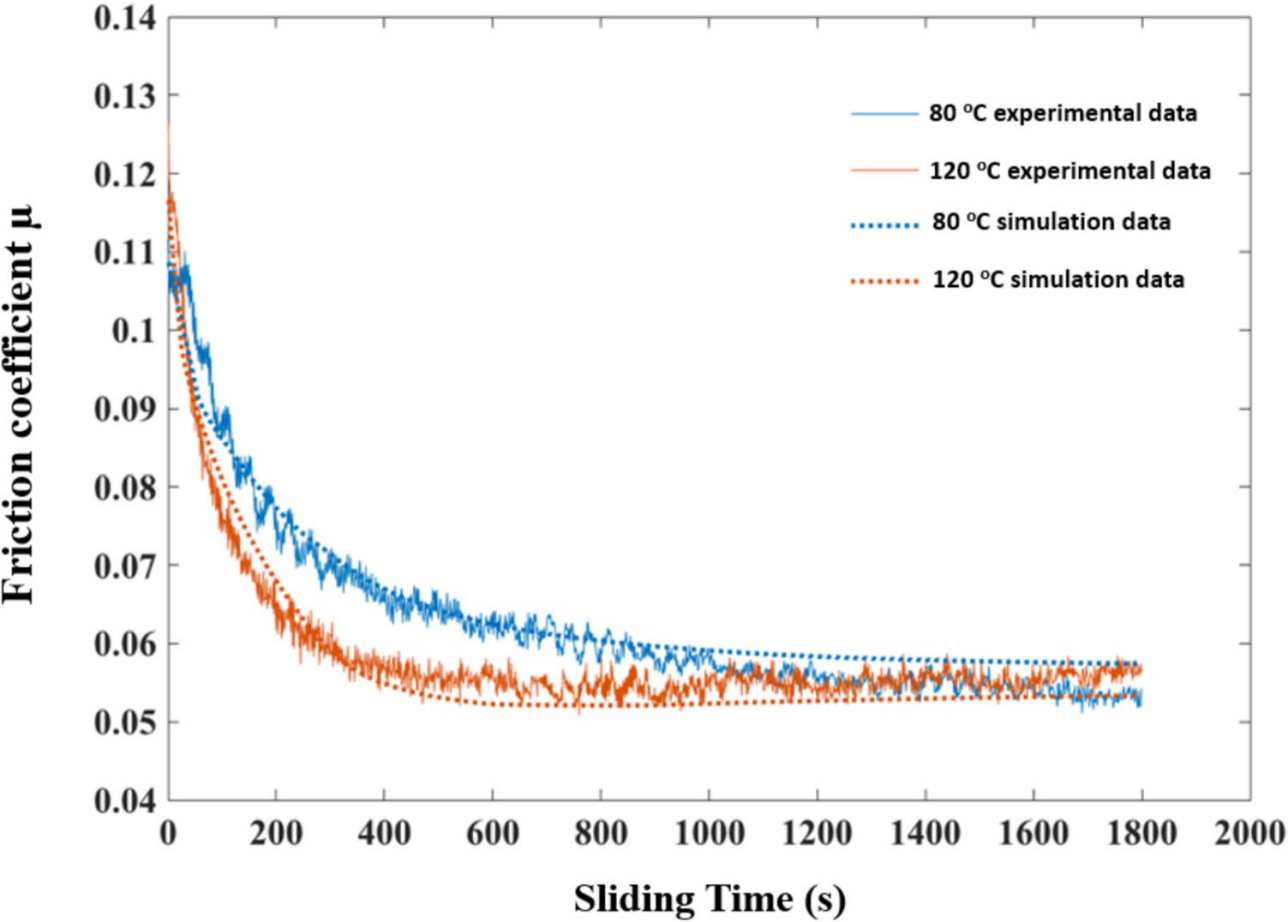
Friction coefficient from simulated Raman intensity maps as a function of sliding time compared with experimental data

Since the Raman intensity maps have been simulated, as shown in Fig. 12 and 13, the resultant friction drops can be predicted based on the MoS_2_ tribofilm dynamics when using a linear relationship between the Raman intensity and the microscopic friction coefficient established in previous work (Eq. 15). The proposed model was validated by comparing the time-resolving friction coefficients from the simulated Raman intensity maps with the friction coefficient data measured, as shown in Fig. 14. There is reasonably close agreement between the simulated friction coefficients and the experimental data at different temperatures. But it should also be noticed that there is some difference in the steady-state friction coefficient values between the simulations and measurements. The formation of ZDDP tribofilm on the surface may have an impact on the friction coefficient as well. This model will be validated using oil containing only MoDTC additive for the future work. Additionally, it can be further developed to capture the growth of ZDDP tribofilm and its subsequent impact on the friction performance.

## Conclusions

A semi-deterministic model capable of predicting friction reduction in boundary lubrication was proposed based on the MoS_2_ tribofilm formation and removal. This study addresses how to collect Raman maps to acquire reliable quantitative analysis of MoS_2_ tribofilms across the wear scar. Four Raman maps with a dimension of 80 × 30 µm^2^ are sufficient to achieve an accepted low sampling error. Then, the average growth rate of MoS_2_ tribofilms was determined using calibrated Raman maps in conjunction with numerical models.

The boundary friction model incorporating tribochemistry was numerically implemented. The simulation results were presented to validate the model, aligning it with published experimental results [25]. This validation spanned from micro-scale Raman maps of MoS_2_ tribofilms to macro-scale friction measurements. The numerical results provided insight into the relationship between tribochemical reactions and asperity contact severity, revealing that MoS_2_ tribofilms are more likely to form in areas of higher contact severity, where the gaps are relatively smaller in comparison to the surface roughness. The developed model effectively simulates both the localised MoS_2_ tribofilm growth and macro-level trend of friction reduction in the boundary lubrication regime. The model can be adapted to a wide range of experimental conditions and various surface geometries.

## Supplementary Information

Below is the link to the electronic supplementary material.

Supplementary file1 (DOCX 2203 KB)

## Data Availability

No datasets were generated or analysed during the current study.

## List of Symbols

*A*: MoDTC additive concentration in the base oil (mol m^−^^3^)
*C*: MoS_2_ species concentration in the tribofilm matrix (mol m^−^^3^)
*k*: Reaction rate (s^−^^1^)
*χ*: Tribo-factor
*C*_*i*_: Constants related to the MoS_2_ growth rate
*T*: Surface temperature (K)
*g*: Gap between the rough surfaces (m)
*σ*: Surface roughness (m)
*α*: Coverage of MoS_2_ tribofilm on the MoDTC/ZDDP tribofilm surface
*I*: Raman intensity (Counts)
*u*_*e*_: Elastic deformation (m)
*p*_a_: Contact pressure (Pa) on the asperity
*τ*_a_: Shear stress (Pa) on the asperity
*E*^***^, *E*_1,2_: Young’s modulus (Pa)
*υ*: Poisson ratio
*H*_*s*_: Hardness of substrate (GPa)
*H*_*T*_: Hardness of MoDCT/ZDDP tribofilm (GPa)
*h*_*T*_: Average thickness of MoDCT/ZDDP tribofilm (m)
*u*_*p*_: Plastic deformation (m)
*q*: Heat flux (W⋅m^−2^)
*μ*: Microscopic friction coefficient
*u*_*s*_: Sliding velocity (m s^−^^1^)
*μ*_*l*_: Low friction coefficient
*μ*_*h*_: High friction coefficient
*ρ*_s_: Density of the solids (kg/m^3^)
*c*_s_: Specific heat of the solids (J/kg K)
*α*_s_: Thermal diffusivity of solid
Δ*T*: Contact temperature (K)
*T*_*b*_: Oil bulk temperature (K)
*t*: Time (s)
