# Supplementary material for: Modelling Friction Reduction Based on Molybdenum Disulphide Tribofilm Formation and Removal in Boundary Lubrication
Source: Tribol Lett. 2025 Mar 17;73(2):46. doi: 10.1007/s11249-025-01981-6 (PMC11914352; doi:10.1007/s11249-025-01981-6)
Supplement: Supplementary file 1 — Supplementary file1 (DOCX 2203 KB) [file 11249_2025_1981_MOESM1_ESM.docx]

| 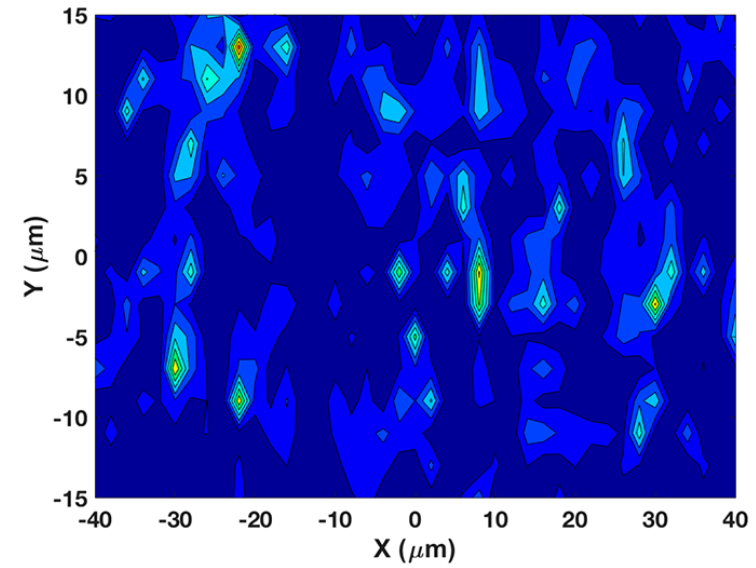 | 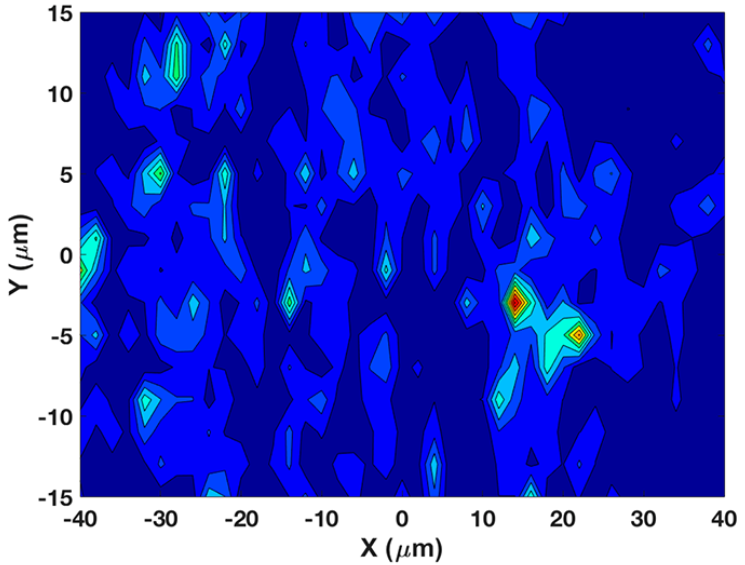 | 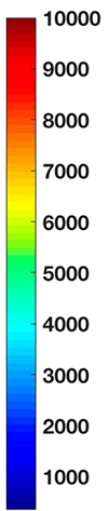 |
| --- | --- | --- |
| (a) 2.5 mins | (b) 5 mins |  |
| 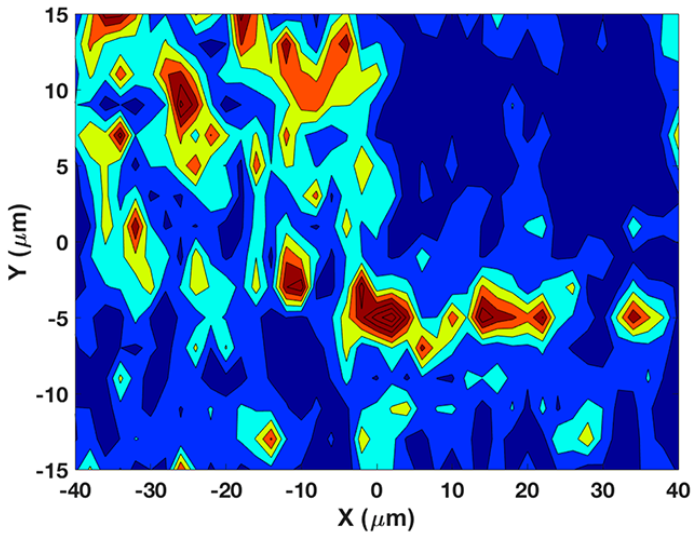 | 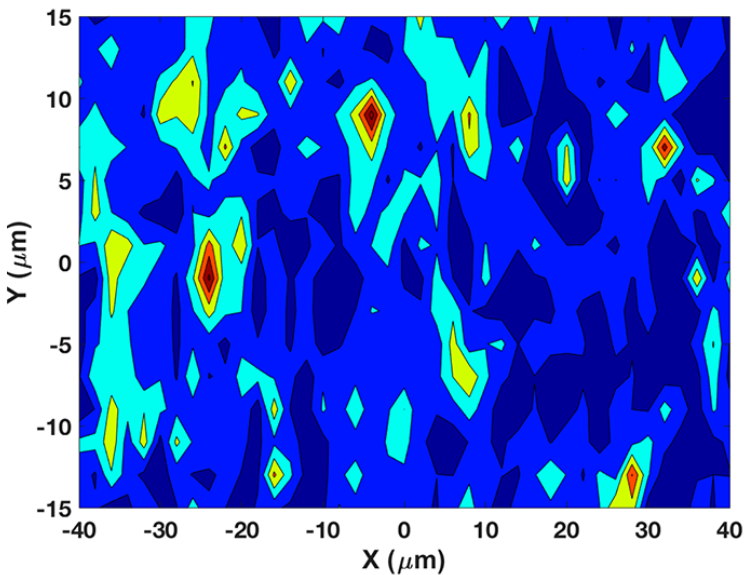 |  |
| (c) 15 mins | (d) 30 mins |  |

**[Supplemental Material](https://us.sagepub.com/en-us/nam/supplementary-files-on-sage-journals-sj-guidelines-for-authors)**

Fig. S1 *Ex-situ* Raman maps of the A1g peak intensity at 80 °C during different periods of rubbing, reprinted from Ref. [25]

| 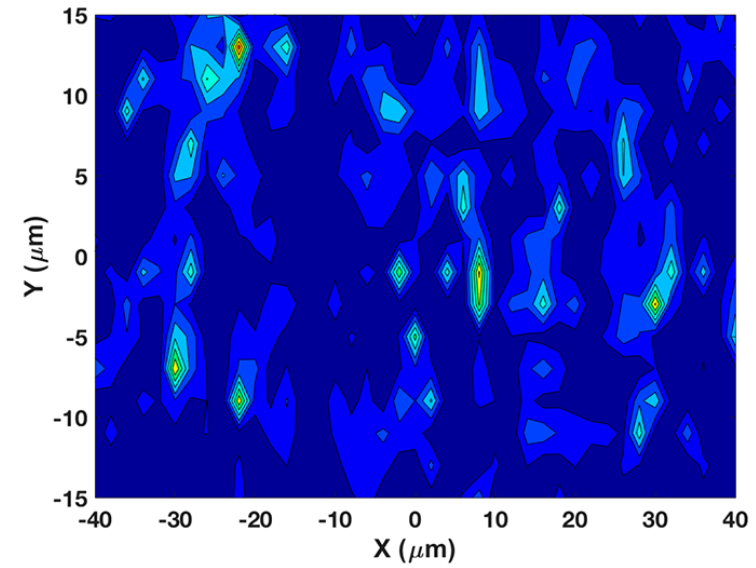 |  | 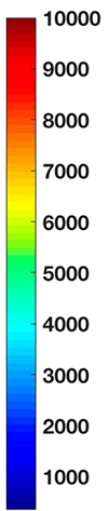 |
| --- | --- | --- |
| (a) 2.5 mins | (b) 5 mins |  |
| 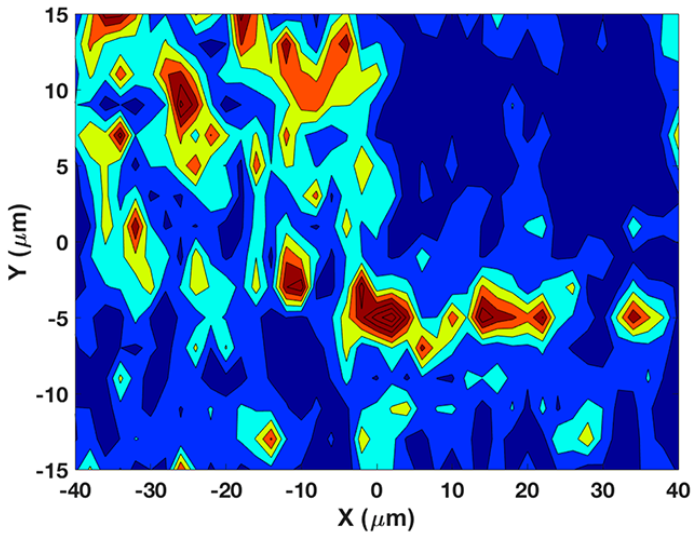 | 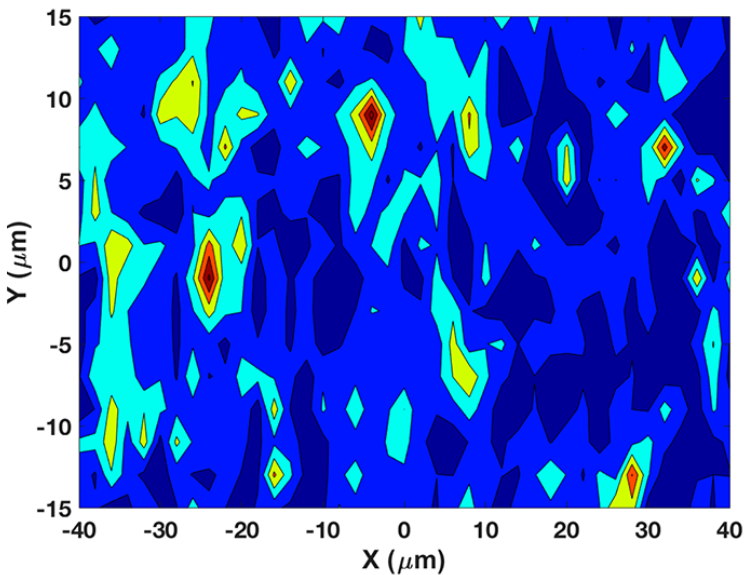 |  |
| (c) 15 mins | (d) 30 mins |  |

| 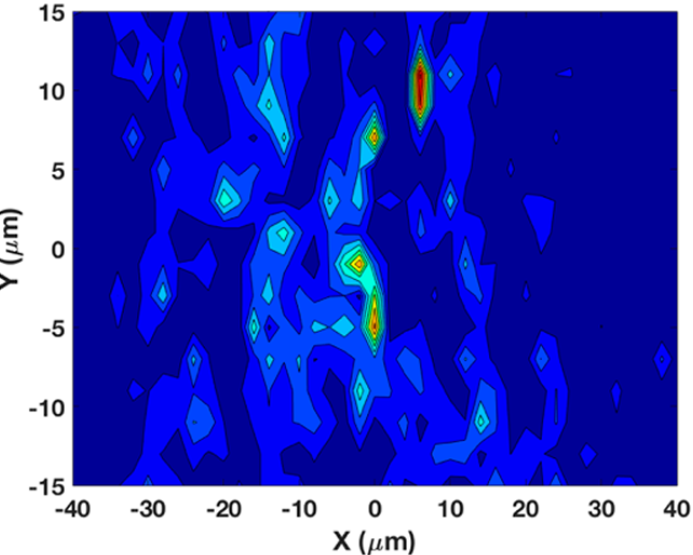 | 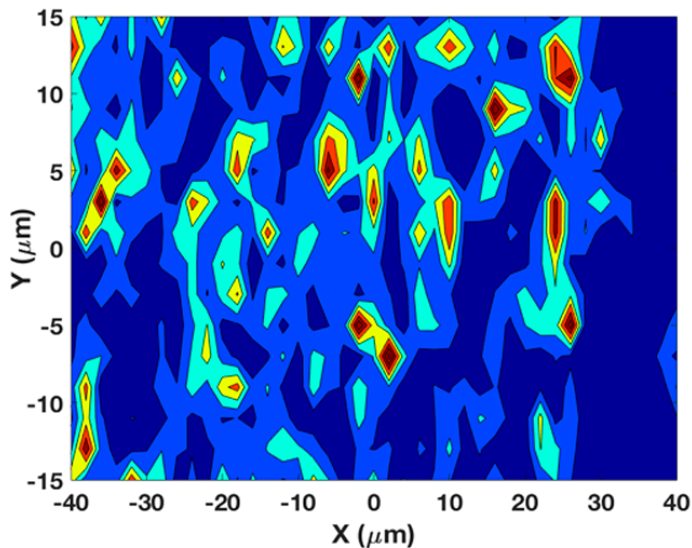 | 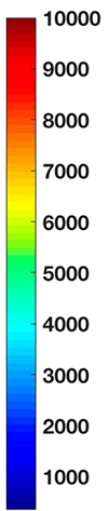 |
| --- | --- | --- |
| (a) 2.5 mins | (b) 5 mins |  |
| 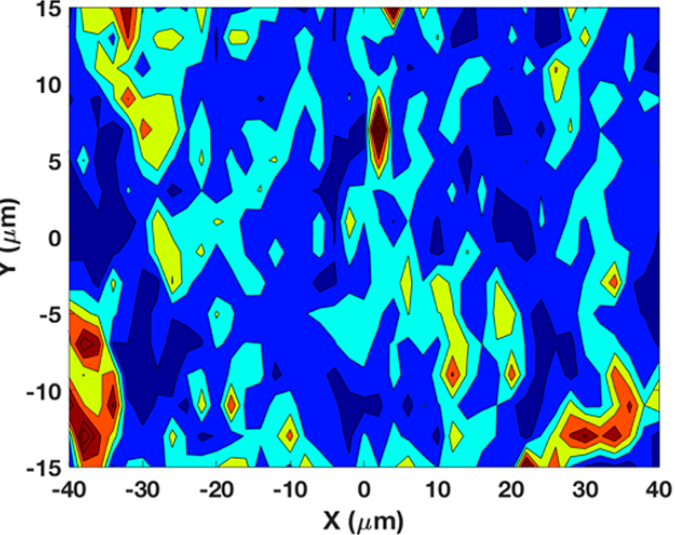 | 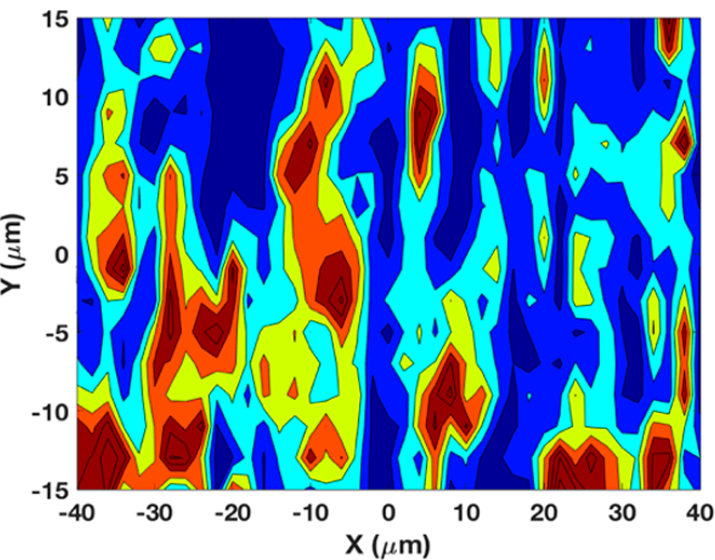 |  |
| (c) 15 mins | (d) 30 mins |  |

Fig. S2 *Ex-situ* Raman maps of the A1g peak intensity at 120 °C during different periods of rubbing, reprinted from Ref. [25]
